# Hyperbolic Discounting: Value and Time Processes of Substance Abusers and Non-Clinical Individuals in Intertemporal Choice

**DOI:** 10.1371/journal.pone.0111378

**Published:** 2014-11-12

**Authors:** Jiuqing Cheng, Claudia González-Vallejo

**Affiliations:** Department of Psychology, Ohio University, Athens, Ohio, United States of America; Middlesex University London, United Kingdom

## Abstract

The single parameter hyperbolic model has been frequently used to describe value discounting as a function of time and to differentiate substance abusers and non-clinical participants with the model's parameter *k*. However, *k* says little about the mechanisms underlying the observed differences. The present study evaluates several alternative models with the purpose of identifying whether group differences stem from differences in subjective valuation, and/or time perceptions. Using three two-parameter models, plus secondary data analyses of 14 studies with 471 indifference point curves, results demonstrated that adding a valuation, or a time perception function led to better model fits. However, the gain in fit due to the flexibility granted by a second parameter did not always lead to a better understanding of the data patterns and corresponding psychological processes. The *k* parameter consistently indexed group and context (magnitude) differences; it is thus a mixed measure of person and task level effects. This was similar for a parameter meant to index payoff devaluation. A time perception parameter, on the other hand, fluctuated with contexts in a non-predicted fashion and the interpretation of its values was inconsistent with prior findings that supported enlarged perceived delays for substance abusers compared to controls. Overall, the results provide mixed support for hyperbolic models of intertemporal choice in terms of the psychological meaning afforded by their parameters.

## Introduction

Psychological research on behavioral control has widely used the intertemporal choice paradigm with results generally demonstrating a preference for immediate, smaller rewards over delayed, larger ones by animals and humans. This result is termed “temporal discounting” and the degree to which individuals discount has been linked to important life consequences such as creditworthiness [1] and indices of health behaviors such as exercise and smoking [2].

Early studies with pigeons showed that it was possible to find a point of indifference between a standard alternative with a constant reinforcement, and a moving alternative for which the contingencies changed depending on the choices of the subject [3]. The adjustment consisted of increasing or decreasing a delay to reinforcement based on the animal's previous choice. This procedure allows for the empirical derivation of a point of indifference between the two alternatives. The magnitude of this indifference point is conceived as a measure of the degree to which a future value is discounted.

Research on substance abuse has used the basic intertemporal choice paradigm to better understand impulsivity. The paradigm reflects the everyday choice that a substance abuser confronts: the drug offers an immediate euphoria (a sooner smaller reward), whereas not taking the drug offers future happiness and healthiness (a delayed larger reward). To date, studies have shown that substance abusers exhibit excessive preference for the sooner reward when compared to the behavior of healthy individuals [4]; hence, they are more impulsive [5]. The basis for the observed group difference is still not fully understood because temporal discounting may result from time distortions, differential time weighting, or from differential subjective valuation processes. Indeed, there is neural evidence suggesting that the evaluation of delayed rewards involves different psychological processes from those of self-control [6].

The mathematical representation of temporal discounting was first depicted by the exponential model, which assumed that value was discounted at a constant rate (delay-independent), and was amount-independent [7]. The behavioral anomaly of dynamic inconsistency-people reverse their time preference when a constant delay is added to both options-poses challenges to this model resulting in further mathematical refinements. In this study we focus on the hyperbolic model, which is the most widely used mathematical description of the discounting phenomenon [7]–[9]

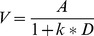
(Equation 1)with V representing the value of the reinforcement delivered after a fixed delay D, A is the amount of the reinforcement, and *k* is a free parameter with greater values indicating greater discounting [7]. This one-parameter model (we refer to it as the base-model in what follows) is able to describe the diminishing value of A as the delay increases and assumes no devaluation of A at D = 0. This model has been useful in representing differences between substance abusers and the non-clinical population via differences in *k*. For example, a meta-analysis showed that a great proportion of studies used the parameter *k* to compare the behavior of substance abusers and healthy individuals [4]. Further, this study showed that on average, the substance abusers exhibited a larger parameter *k* compared to healthy people. More recently, a field study on social cooperation [10] used *k* as a measure of individuals' impatience and examined its relationship to the likelihood of inflicting punishments on free riders when playing a one-shot, public good game with punishment. Results showed that punishment by highly cooperative individuals was carried out by the more patient, whereas punishment by low cooperators was carried out by impatient individuals. These results suggest interesting relationships between self-control as measured by *k*, and the application of sanctions in social domains.

In the present study, we focus on further exploring the differences between discounting by healthy and substance dependent individuals via modeling and parametric analyses of models based on Equation 1. In particular, we seek to examine group differences in terms of valuation versus time processes. We note that we restrict our models to functions that have been widely used to describe the behavior of substance abusers. Hence, models such as Laibson's quasi-hyperbolic model [11], [12] or forms based on the exponential model [13] are not included in the current study.

### Valuation Processes

A large body of literature contends that payoffs are subjectively represented within each individual (going back to at least to Bernoulli [14]), and most theories, including prospect theory, contend that the subjective value is non-linearly related to the nominal value [15]. Lowestein and Prelec reviewed the economic utility and the discounted utility functions when describing anomalies in intertemporal choice and proposed a more general discounting model based on both the hyperbolic model and a value function similar to prospect theory's [16]. We follow this work and take a non-linear valuation function in accord with Steven's law to represent subjective value. In essence Steven's law captures the relationship between physical intensity and perceived intensity via a power function [17]. The following equation represents the addition of a valuation function to the base-model, where V, *k*, A, and D are as previously defined.
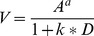
(a–model)


The added parameter *a* (0<*a*) adjusts the perceived valuation of the amount A>0; parameter *a*<1 represents diminishing subjective value, which is commonly assumed in utility models. It is possible that average group differences in discounting between substance abusers and non-clinical participants stem from different valuation functions with substance abusers having smaller *a*, everything else constant.

An interesting phenomenon in substance abusers is that they are readily willing to give up money in order to get drugs; they are likely to purchase the drugs right after they get their paychecks, and presumably before they are in the craving stage [18]. Thus, money may be conceived as a secondary reinforcer with its value depending on how soon the cash is exchanged for drugs. Because exchanging the money for drugs requires time, money may be further discounted; that is, substance abusers may perceive an additional delay between receiving the secondary reinforcer (money) and the primary reinforcer (drugs). The *a*-model may be rewritten into a representation that elucidates this property: first multiplying the right side of the equation by A^1-a^/A^1-a^, we return to A in the numerator, and the denominator becomes (c+k*D). We label this model as the *c*-model:
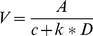
(c–model)with *c* = A^1-a^ and with *a* = 1 – ln(*c*)/ln(A) for a constant A. Values of *c*>1 imply *a*<1 for a constant A>1. In this model the subjective value of A dampens when *c*>1 (for A>1), everything else constant, thus representing the property of marginally decreasing subjective value. Re-expression of the denominator in the *c*-model results in: (1+k*(*d*+D)) with *c* = 1+k*d. Here we take the addition of the quantity *d* to the delay D to stand for the additional time that takes to exchange the money into drugs (we thank an anonymous reviewer for this expression). This representation makes the secondary nature of money more evident as an additional subjective delay slows down receiving a primary reinforcer. Thus, it is possible that substance abusers experience elongated delays that further reduce the subjective value of money, compared to controls.

### Time Processes

The excessive preference of substance abusers towards the sooner, smaller reward may be due to their abnormal time perception [19]. For example, it was found that stimulant-dependent individuals tended to overestimate a period [20]. We take two commonly used models in the literature to explore the time related processes. The first one was derived by Rachlin and contains a power function to represent subjective time [8],
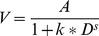
(Rachlin – model)with parameter *s* indicating the subjective sensitivity to delay; 0<*s*<1 implies subjective values of D that are smaller than D, or a diminished time sensitivity. The finding that subjective time estimation changed less than corresponding changes in objective time [21] provided support for parameter values in this range. In this experiment, participants were given a 180 mm line with end-points labeled “very short” and “very long” to indicate how long they perceived a delay to be. It was found that the subjective times that corresponded to 3 months, 1 year, and 3 years were 105.9 mm, 131.3 mm, and 140.0 m, respectively. Thus, the perception of time was negatively accelerated with objective time. For values of *s*>1, time is enhanced (such as the feeling that time stretches when waiting in a long queue). A smaller V results when *s*>1, all else equal, thus resulting in greater discounting of A; the opposite pattern holds when *s*<1.

The second model in this class is the one advanced by Green and colleagues [22], [23]. In this model, the parameter *s* scales the sensitivity toward delay in a more global manner. Myerson & Green (1995) [24] indicated that the *s* parameter is to reflect individual differences in scaling amount and time and was defined as the ratio of two exponents that scale time and amount, respectively. The paper [24] further stated that “…the exponent *s* might be expected to remain constant when the same individual confronts different choice situations.” (p. 274).
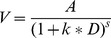
(Green – model)


In the equation above, at a fixed *s*, greater *k* results in greater discounting. Similarly, for a constant *k*, a greater *s* results in greater discounting.

### Function Form Analysis

The models under consideration stem from different psychological assumptions about the process of discounting. Needless to say, they all conserve the parameter *k* as a means of representing time discounting via a weight on delay; however, because the function forms do vary as the result of the other parameters (namely *c* and *s*), the same *k* will not have the same impact on V across models and this is important from the perspective of interpreting values of *k*. Thus, to the extent that other parameters vary across groups, they may shed light on psychological differences stemming from valuation or time perception, or both. Note that all models reduce to the base-model when *a* = *c* = *s* = 1.

In this section we briefly demonstrate the changes in the discounting curves defined by the two-parameter models reviewed. We show that the models do not always overlap, but model differences may be hard to detect depending on the conditions of testing. In particular, because in the limit the functions go to 0 as D approaches infinity, larger values of D present more challenging testing situations for model discrimination. In addition, the amount A affects the valuation directly not only influencing the intercept but also affecting the slope (i.e., amount of value decrease per unit of time increment). We adopt different magnitudes of the parameter values to show their effect on the subjective value of A (i.e., V). In addition, because the *a*- and *c*-models are equivalent, we only present results of the *c*-model.

**Figure 1:**
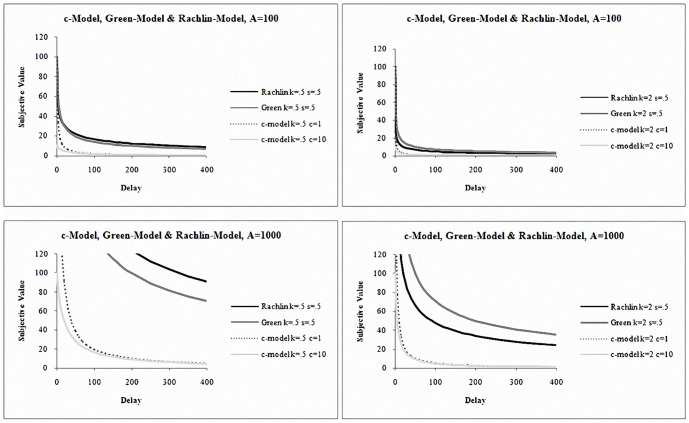
Subjective Value of A from *c*-, Rachlin- with parameters *k* = 0.5 and 2; *c* = 1 and 10; *s* = 0.5. A = 100 and 1000.

Figure 1 plots V for *c-*, Rachlin-, and Green-models for two levels of *k* (*k* = 0.5 and *k* = 2), two levels of *c* (*c* = 1 and *c* = 10), and one level of *s* (*s* = 0.5). The figure also displays the functions at two levels of A (A = 100 and A = 1000). First, Figure 1 shows the greater discounting with larger values of *k* in all models. Furthermore, the behavior of the Rachlin- and Green-models are fairly similar for a constant s = 0.5 with greater differentiation when *k* is greater. The *c*-model produces greater discounting with a larger *c*, but the effect of *k* is important. When *k* is large, the *c* parameter produces little change in discounting as a result of a rather large change in *c* (going from *c* = 1 to *c* = 10). When *k* is small, on the other hand, the change in *c* is more pronounced. Both the base-model (*c* = 1) and the *c*-model with larger *c* tend to produce greater discounting in all conditions, but the differences are more salient with greater A. Furthermore, the change in value per unit of time is larger at greater A. This is particularly clear when *k* is large (right hand-side panels). By 100 units of delay D, the subjective value of A = 100 is nearly zero for all models; this is not so for A = 1000.

**Figure 2:**
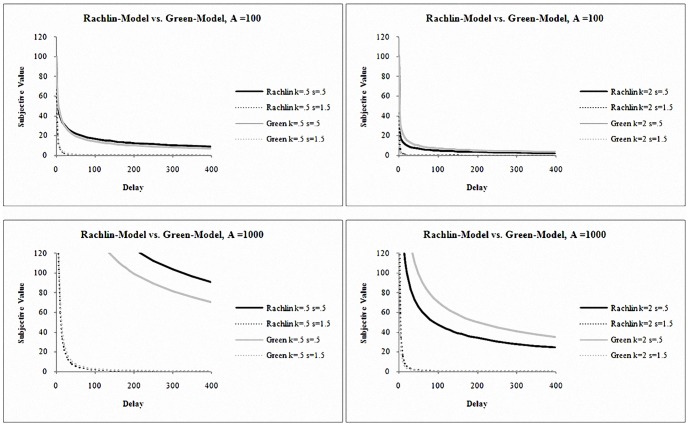
Rachlin- and Green-Models with varying parameters *s* = 0.5 and 1.5; *k* = 0.5 and 2. A = 100 and 1000.

In order to explore the effects of the *s* parameter in the Rachlin- and Green-models, Figure 2 shows plots that vary *s* (0.5 and 1.5), and *k* (0.5 and 2) and the size of A (100 and 1000). In Figure 2, greater discounting is observed with greater *s* (dotted lines). The difference between the models is small in most situations but they have less overlap with smaller *s* (solid lines). When *s*<1 (solid lines) and *k*<1, Green-model produces greater discounting than Rachlin-model and the reverse is true when k>1; however, the effect is more easily observed at greater A (bottom panels) and in some range of D. As D increases, nevertheless, all models make similar predictions as they approach 0 in the limit. Figure 2 also shows how the *s* parameter regulates the effect of *k*. A relatively small *s* can counteract the discounting effect of a large *k* in both the Rachlin- and Green-models (comparing solid to dotted lines in Figure 2). Thus *s* tends to regulate the inflection and slope of the curves whereas *k* is more closely related to the general base level of discounting.

As seen in Figures 1 and 2, the ability to distinguish the models depends greatly on the levels of A and D for any given set of parameter values. In particular, the slopes of the functions (measuring the change in value per unit of time) vary with changes in the different conditions. Furthermore, expanding the time horizon diminishes model differences. Interestingly, the models are silent with regards to the actual units of D. If D were days in Figure 2 at A = 100, *k* = 2, the models predict similar subjective values of A beyond 100 days (i.e., approximately 3 months, or ¼ year). If D were weeks, on the other hand, the models predict similar behavior beyond 100 weeks, 24 months, or approximately 2 years.

### Overview

The goal of this study is to use the mathematical models presented and examine the factors underlying discounting of substance abusers (SA) and non-clinical (NC) people. More specifically, we use available behavioral data for both SA and NC with the aim of elucidating the different psychological processes underlying discounting with a modeling comparison approach. This approach analyzes both model fit and parameter interpretability. In particular, we set to understand if the basis for group differences resides in value versus time processes. We also examine the behavior of model parameters in terms of describing a well known contextual effect that shows changes in discount tendencies as a function of payoff size-*the magnitude effect*.

## Methods

### Data Selection

Previous research on intertemporal choice used aggregate (i.e. group) data or individual data to test model fitting (e.g. [8], [25]). Because mathematical representations of behavior on the aggregate level may not reflect individual level behavior [26], [27], we sought data that would allows us to fit the models at the level of each individual in each condition tested (i.e. indifference curve level). Data were obtained from published and unpublished sources (see Table 1) and the study fitted the models to *N* = 471 indifference curves.

**Table 1:** Demographic Information of Participants and Experimental Design.

	Basic demographic information of participants					General information of experimental design		
Study	N*	Mean age	Gender	Mean education	Substance abusers or non-clinical participants	Rewards	Delays	Staircase or titration
Bickel, Yi, Landes, Hill & Baxter (2010) condition A [28]	27	38.6	20 males and 7 females	12.2	20 cocaine users, 5 methamphetamine users and 2 mixed users	Real $100	1 day, 1 week, 1 month and 6 months	Staircase
Bickel, Yi, Landes, Hill & Baxter (2010) condition B and C [28]	54	Same as above				Hypothetical $100 and hypothetical $1,000, respectively	1 day, 1 week, 1 month, 6 months, 1 year, 5 years and 25 years	Same as above
Odum, Madden, Badger & Bickel (2000) [29]	17	32.7	11 males and 6 females	12.8	Opium (non-needle sharing)	Hypothetical $1,000	1 week, half a month, 1 month, 6 months, 1 year, 5 years and 25 years	Staircase
Cheng, Lu, Han, Gonzalez-Vallejo & Sui (2012) heroin abusers [30]	112	34.1	41 males and 15 females	10.2	Herion abusers	Hypothetical 200RMB and 50,000RMB (Chinese currency)	1 day, 1 week, 1 month, 6 months, 1 year, 5 years, 10 years and 20 years	Titration
Cheng, Lu, Han, Gonzalez-Vallejo & Sui (2012) controls [30]	112	36.0	Same as above	10.6	non-clinical participants	Same as above		
Estle, Green, Myerson & Holt (2006, Exp 1) [22]	40	19.5	7 males and 13 females	undergraduate	non-clinical participants	Hypothetical $200 and $40,000	1 month, 6 months, 1 year, 3 years, 5 years, 10 years and 20 years	Titration
Estle, Green, Myerson & Holt (2006, Exp 3) [22]	81	19.2	15 males and 12 females	undergraduate	non-clinical participants	Hypothetical $100, $20,000 and $60,000	1 month, 6 months, 1 year, 3 years, 5 years, 8 years, 12 years, and 20 years	Titration
Cheng & Gonzalez-Vallejo (unpublished data)	28	NA	NA	undergraduate	non-clinical participants	Hypothetical $600	1, 10, 30, 50, 100, and 150 days	Staircase

*: The number of actual discounting curves in each condition that was obtained from different individuals.

For data selection, we used three inclusion criteria that were pertinent to our goals. 1) The studies had adopted a typical intertemporal choice task that either used staircase procedure or titration procedure. These studies asked participants to make repeated choices between an immediate, smaller reward, versus a delayed larger reward in order to derive a point of indifference that reflects the subjective present value of a future amount. With the staircase procedure, the immediate magnitude was presented in a staircase manner with same increment while the delayed magnitude was fixed. With the titration procedure, the immediate magnitude was adjusted based on participants' previous responses so that the immediate magnitude gradually approached the subjective value of the delay magnitude. With a series of indifference points assessed at different delays, the models could be fitted. 2) The study had fitted a hyperbolic model. This allowed us to begin with data that were feasible for our aim of model comparison. 3) The studies included either substance abusers (SA) or non-clinical participants (NC).

Based on these criteria and a literature search, we contacted seven authors who had several publications and three of them provided us with the data from those publications (the data were chosen by those authors). In addition, we included data from our lab (one published and one unpublished set). We note that our inclusion criteria was not exhaustive of all studies on temporal discounting, because our aim was to both have a uniform set of studies (with similar methodologies) and enough data so that individual model fitting for both SA and NC was possible.

Table 1 details the characteristics of the studies [22], [28]–[30] included in the present manuscript. Each study is described in terms of number of participants, age, gender, education level and drug type (if substance abusers). In describing each experimental design, we included the nature of the rewards (real or hypothetical), the magnitude of the rewards, the delays associated with the rewards and procedure of the task (staircase or titration). It should be noted again that the sample sizes described are not the number of participants. In some studies, one participant completed the temporal discounting tasks in more than one condition (e.g. different magnitudes). Therefore, sample size here indicated the number of actual discounting curves in each condition that was obtained from each of the different individuals. In other words, model fitting was performed at the level of each person's curve. Fitting at discounting curve level allowed us to test if a parameter was able to capture the magnitude effect by comparing this parameter between different magnitude conditions within a same group of participants.

In order to easily identify the different studies in the text of this manuscript we label them using the authors' names as found in Table 1 (from top to bottom): Bickel A, Bickel B, Bickel C, Odum, Cheng Heroin small, Cheng Heroin large, Estle 1 small, Estle 1 large, Estle 3 small, Estle 3 medium, Estle 3 large, Cheng control small, Cheng control large, and OU. The reader can easily find the testing conditions (e.g., whether the study was done with SA or NC; the size of the payoffs, etc.) by referring to Table 1. As seen in Table 1, there are a total of 14 studies.

### Model fitting and Parametric Analyses

Indifference points were directly obtained from the studies. We note that in model fitting we maintained the time units used by the authors of the original studies. For example, although in [22] some delays were presented in years to participants the delays were transformed to months when fitting the Green-model by the authors. We followed the same procedure so that the delay units in our study were the same as those in the original studies. All models were fitted via non-linear regression on individual level using SPSS. The estimated parameters and goodness of fit results were checked for consistency, whenever possible, by comparing them to the results of the original studies when provided by the authors (this was the case for Odum [29]; and for Estle 1 and Estle 3 [22]). We compared the models by focusing on *R^2^*, but importantly, estimated parameters were examined with regards to meaningfulness because models may fit data with nonsensical values. Further SA and NC groups were compared in terms of their parametric representations.

## Results

### Goodness of Fit Analyses

Table 2 and Table 3 list median *R^2^* for all models in each study separating for studies having SA (Table 2) and for those having NC (Table 3). In each study, but Bickel condition B, the *R^2^* of base-model was lower than that of the other models, Wilcoxon Signed Rank Z≥3.408, *p*-values <.01. In Bickel condition B, there was no difference in *R^2^* between base-model and *c*-model, *p*-value>.1. However, base-model fitted more poorly when compared to Rachlin- and Green-models in this study, Wilcoxon Z≥3.629, *p*-values <.01. The *R^2^* differences between the base (one parameter) and the other models (two parameters) were not surprising, however, as a model with more parameters was expected to fit better and in general the base-model fitted well (median *R^2^* = .881 across all studies).

**Table 2:** Median R^2^ for SA Group in Each Study.

Study	Bickel A	Bickel B	Bickel C	Odum	Cheng Heroin	Cheng Heroin
Magnitude	100	100	1000	1000	200	50000
Base-model	.000 (.88)	.753 (.90)	.471 (.90)	.777 (.81)	.855 (.78)	.829 (.77)
*c*-model	.817 (.76)	.753 (.90)	.840 (.50)	.921 (.28)	.959 (.05)	.884 (.09)
Rachlin-model	.700 (.66)	.882 (.40)	.853 (.57)	.940 (.11)	.964 (.03)	.967 (.04)
Green-model	.848 (.89)	.855 (.55)	.859 (.68)	.945 (.16)	.941 (.06)	.963 (.08)

Note: Numbers in parentheses are the interquartile range.

**Table 3:** Median R^2^ for NC Group in Each Study.

Study	Estle 1	Estle 1	Estle 3	Estle 3	Estle 3	Cheng Control	Cheng Control	OU
Magnitude	200	40000	100	20000	60000	200	50000	600
Base-model	.922 (.26)	.898 (.11)	.884 (.11)	.844 (.28)	.881 (.31)	.923 (.23)	.930 (.09)	.921 (.15)
*c*-model	.956 (.17)	.922 (.11)	.930 (.23)	.895 (.16)	.912 (.43)	.980 (.03)	.979 (.03)	.952 (.05)
Rachlin-model	.962 (.08)	.939 (.09)	.946 (.18)	.921 (.18)	.947 (.30)	.989 (.02)	.981 (.03)	.978 (.04)
Green-model	.949 (.07)	.949 (.07)	.938 (.17)	.901 (.20)	.948 (.23)	.981 (.04)	.975 (.04)	.964 (.05)

Note: Numbers in parentheses are the interquartile range.

To further test whether adding a parameter to the base-model was useful, we compared the models using a statistics that penalizes model for being over-parameterized. Schwarz Bayesian Criteria (*SBC*) is a measure of model selection derived by modifying the Bayesian information criterion [31]. It reflects the posterior probability of a specific model being the true model based on the observed data. SBC makes a trade-off between variance that can be accounted for and the complexity of the models. A model with smaller SBC is favored because it explains sufficient variance (compared to competing models) in a relative concise manner; if models provide equal fit from the perspective of variance accounted for, *SBC* penalizes the model with more parameters. *SBC* was computed for each model in each study by first obtaining the median indifference points across participants, then fitting the models to these values. In each case, *SBC* = *n* * ln(SSE/*n*) +*p* * ln(*n*), where *n* was the number of median indifference points corresponding to the number of time delays, SSE was the residual variance, and *p* was the number of parameters. Results showed that *SBC* from base-model was larger than *SBC* from any two-parameter model in each study. Across all the studies, mean *SBC* for base-model, *c*-model, Rachlin-model and Green-model were 60.2, 50.9, 46.8, and 49.2, respectively. Thus, the Rachlin-model fitted best from this perspective.

We proceeded to compare the median *R^2^* values between two-parameter models using the Wilcoxon Signed Rank Z test. First, Rachlin- and Green-models did not show a difference in 10 out of 14 studies, *p*-values>.1. In four conditions in [30], Rachlin-model fitted better, *p*-values <.05. Second, Rachlin-model was superior to the *c*-model in 8 out of 14 studies (Bickel B, Cheng heroin large, Estle 1 large, Estle 3 all three magnitude conditions, Cheng control small, and OU), *p*-values <.05, and marginally better in two studies (Cheng heroin small and Estel 1 small), *p*-values <.1. When comparing *c-* and Green-models, the Green-model was significantly better in 6 out of 14 studies (Bickel B, Cheng heroin large, Estle 1 large, Estle 3 all three magnitude conditions), *p-*values <.05, and marginally better in one study (OU), *p*-value <.1. Hence, the Rachlin-model tended to provide better fits than the *c*-model, but only slightly better fits than the Green-model. This is consistent with the simulation results found in Figures 1 and 2 showing that the Rachlin- and Green-models tend to overlap.

We further compared the median *R^2^* of the two-parameter models for SA and NC, respectively, across all of the studies. We note that the studies were not combined in this analysis as each person's set of data was fitted individually. To the extent that a model was fitted at this micro-level, all specifics of the study were assumed to be reflected in the estimated parameter values. Table 4 displays the median *R^2^* for each model in SA and NC.

**Table 4:** Median R^2^ Within SA and NC.

	SA	NC
*c*-model	.906 (.192)	.960 (.070)
Rachlin-model	.953 (.119)	.971 (.058)
Green-model	.937 (.133)	.964 (.073)

In both populations, Rachlin-model had the highest median *R^2^*. Green-model took the second place and *c*-model had the lowest fit among the three, all *p*-values <.01, but again all models demonstrated excellent fit (median *R^2^*>.93).

**Figure 3:**
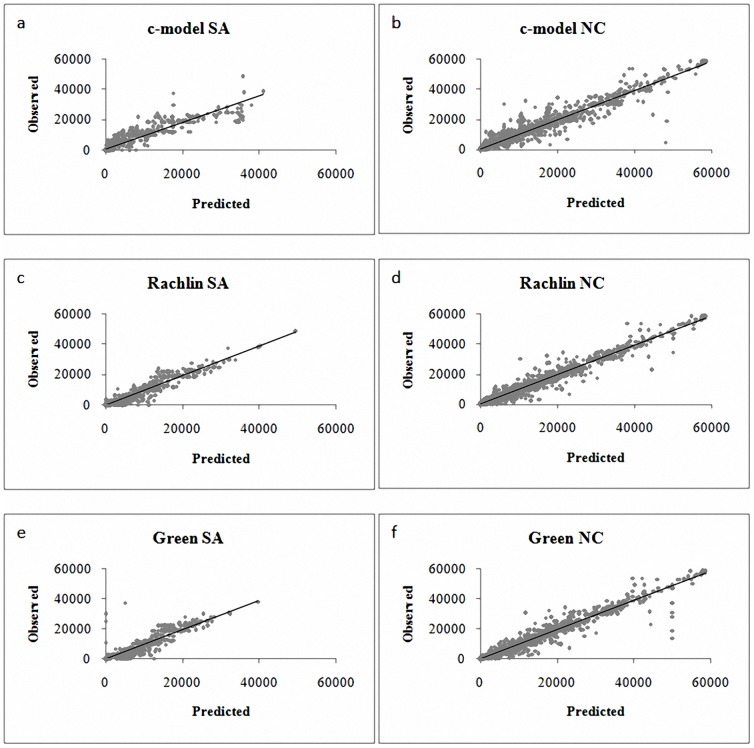
Scatter Plots of Predicted and Observed Indifference Points for Each Model in each SA and NC Groups.

Another way of examining the predictive validity of the models was to perform a linear regression with predicted and observed values as the predictor and the criterion, respectively. A perfect prediction would result in a line with slope equal to one and an intercept equal to zero. This analysis was conducted using each person's observed and predicted values and Figure 3 depicts the scatter plot of predicted and observed indifference points and the linear fits for each model in SA and NC. Table 5 provides *R^2^*s, slopes, and intercepts of the linear fits in Figure 3. In both populations, the linear fit of Rachlin-model was closest to the optimal values; Green and *c*-models followed (we note that we kept outliers in this analysis so that all models had the same number of observations). Taken together, all analyses indicated that Rachlin-model provided the best fit.

**Table 5:** Linear Regression of Predictive Validity of Models in SA and NC.

	slope	intercept	*R^2^* of linear fit
*c*-model SA	.883	592.50	.873
Rachlin-model SA	.979	−48.846	.942
Green-model SA	.978	−99.155	.883
*c*-model NC	.969	533.763	.940
Rachlin-model NC	.979	27.628	.969
Green-model NC	.972	54.236	.957

### Analyses of Estimated Parameter Values

As is well known in the mathematical psychology literature, models may fit well but with non-interpretable parameters. Therefore, analysis with modeling must be concerned not only with fitting, but also with the meaningfulness of the parameters obtained [32]. We proceeded to examine outliers in the estimated parameters in order to gain a better sense of the performance of the models. We looked for very strange parameter values that could not be interpretable. For example, a participant had estimated parameters of *k* and *s* from Green-model equal to 5.6×10^11^ and.0711, respectively. The extremely large value for *k* in this case implies an indifference curve that drops immediately after the intercept A and remains essentially flat in the range of values of D studied. Instead of adopting conventional mean ± 3SD to detect outliers, we used this rather conservative approach to select outliers (i.e., selecting extremely large or extremely small estimated values), and found that across all studies, only 11 cases (out of 472, or 2.33%) were found to have outliers for *c*-model. The Rachlin-model had 4 cases (0.85%); and Green-model had 22 cases (4.66%). The cases identified by this procedure also had very low *R^2^*s (*R^2^*≤.201). Because the outlying cases constitute a very small percentage of the data, we keep them in all of the analyses except when focusing on the interpretation of mean parameter values as will be noted. We performed all previously reported group analyses by taking the outliers out and the conclusions remained unchanged.

The analyses with SBC revealed that the two-parameter models improved model fitting beyond simply increasing model complexity. We proceeded to test the specific values of the estimated parameters *c* and *s* in order to better understand the contribution they make to the base-model because when *c* (*a*) = *s* = 1 the models reduce to the base-model. Table 6 shows the median and mean values of the parameters for each model in each study. We note that values in Table 6 were calculated without the outliers. Doing so had little impact on medians, but was important in interpreting the mean values.

**Table 6:** Median and Mean Values of Parameters in Each Study.

		*c*-model		Rachlin-model		Green-model	
		*k*	*c*	*k*	*s*	*k*	*s*
Bickel A^SA^	Median	.004 (.03)	1.133 (.50)	.096 (.43)	.257 (.60)	1.251 (6.9)	.273 (.43)
	Mean	.020 (.03)	1.589 (1.3)	.456 (1.0)	.474 (.63)	15.14 (29.4)	1.651 (4.68)
Bickel B^SA^	Median	.002 (.009)	1.092 (.20)	.041(.13)	.486 (.55)	.040 (.613)	.252 (.44)
	Mean	.058 (.009)	2.839 (8.46)	1.468 (7.2)	.603 (.51)	4.564 (10.9)	.307 (.27)
Bickel C^SA^	Median	.002 (.009)	1.092 (.38)	.066 (.24)	.423 (.49)	.178 (1.6)	.288 (.59)
	Mean	.030 (.08)	1.330 (.83)	.269 (.67)	.604 (.91)	5.112 (16.1)	.399 (.36)
Odum^SA^	Median	.013 (.641)	1.067 (.32)	.259 (2.4)	.980 (1.6)	3.97(10.7)	.175 (85)
	Mean	2.41 (6.81)	1.511 (1.53)	3.089 (7.8)	1.438 (1.5)	10.27 (16.2)	.483 (.50)
Cheng Heroin small^SA^	Median	.008 (.007)	1.276 (.35)	.117 (.24)	.512 (.27)	.122 (.72)	.333 (.17)
	Mean	.010 (.01)	1.527 (.91)	.270 (.43)	.556 (.19)	1.965 (5.25)	.382 (.20)
Cheng Heroin large^SA^	Median	.002 (.002)	1.151 (.23)	.051 (.11)	.520 (.23)	.047 (.23)	.286 (.13)
	Mean	.002 (.001)	1.292 (.39)	.153 (2.7)	.539 (.32)	.754 (3.25)	.260 (.12)
Estle 1 small^NC^	Median	.054 (.10)	1.058 (.39)	.193 (.42)	.867 (.56)	.268 (1.30)	.767 (.71)
	Mean	.109 (.19)	1.202 (.40)	.265 (.42)	.878 (.47)	1.122 (2.5)	.694 (.41)
Estle 1 large^NC^	Median	.009 (.02)	1.032 (.07)	.024 (.07)	.774 (.37)	.035 (.15)	.419 (.76)
	Mean	.040 (.09)	1.046 (.13)	.070 (.11)	.864 (.38)	.150 (.266)	.920 (1.7)
Estle 3 small^NC^	Median	.051 (.17)	1.192 (.38)	.205 (.20)	.641 (.43)	.549 (1.15)	.439 (.52)
	Mean	.114 (.16)	1.270 (.37)	.316 (.32)	.705 (.27)	1.106 (.25)	.694 (.63)
Estle 3 medium^NC^	Median	.007 (.02)	1.026 (.08)	.020 (.03)	.871 (.45)	.027 (.12)	.550 (.87)
	Mean	.013 (.02)	1.056 (.10)	.037 (.06)	.834 (.38)	.508 (1.84)	3.76 (13.3)
Estle 3 large^NC^	Median	.006 (.02)	1.019 (.13)	.012 (.05)	.846 (1.04)	.019 (.27)	.732 (37.4)
	Mean	.013 (.01)	1.072 (.16)	.066 (.10)	1.004 (.61)	.344 (1.2)	17.40 (26.1)
Cheng Control small^NC^	Median	.004 (.002)	1.040 (.07)	.012 (.02)	.808 (.20)	.009 (.01)	.653 (.22)
	Mean	.005 (.004)	1.088 (.16)	.043 (.01)	.789 (.17)	.187 (.95)	.586 (.23)
Cheng Control large^NC^	Median	.001 (.0004)	1.061 (.06)	.008 (.01)	.663 (.20)	.005 (.005)	.381 (.19)
	Mean	.0009 (.0005)	1.067 (.06)	.014 (.02)	.753 (.27)	.008 (.06)	.535 (1.2)
OU^NC^	Median	.008 (.01)	1.036 (.09)	.014 (.04)	.844 (.44)	.008 (.02)	.525 (1.61)
	Mean	.011 (.01)	1.056 (.08)	.041 (.06)	.832 (.30)	.079 (.23)	3.045 (7.2)
SA	Median	.003 (.009)	1.157 (.34)	.086 (.26)	.503 (.38)	.120 (4.07)	.282 (.31)
	Mean	.346 (2.74)	1.645 (3.06)	3.078 (9.8)	.419 (1.36)	.754 (3.9)	.958 (4.7)
NC	Median	.004 (.015)	1.043 (.95)	.016 (.05)	.771 (.34)	.012 (.09)	.465 (.54)
	Mean	.041 (.23)	1.099 (.21)	.355 (1.35)	1.742 (6.2)	.086 (.19)	.827 (.35)
Total	Median	.004 (.01)	1.071 (.21)	.032 (.14)	.646 (.40)	.024 (.16)	.347 (.44)
	Mean	.173 (1.82)	1.278 (1.99)	.382 (2.61)	.725 (.52)	.468 (1.37)	.865 (3.24)

Note: Numbers in parentheses are IQR and standard deviation for median and mean values, respectively. SA: median and mean of parameter values computed across all cases in studies having substance abusers; NC: median and mean of parameter values computed across all cases in studies having non-clinical participants. The Total has the median and mean computed across all cases and studies. When SA and NC are superscripts, they indicate whether the study contains substance abusers or non-clinical people.

As shown in Table 6, in all studies the median and mean *c* were greater than 1. In terms of the *c*-model's alternative representations, this implies that *a*<1 (or *d*>0; see *c*-model descriptions). The median and mean *s* were smaller than 1 with two exceptions for mean Rachlin-*s* and four exceptions for mean Green-*s*. Table 7 further displays the percentage of the parameter values that were above (for *c*) or below (for *s*) the default value of 1 in each study.

**Table 7:** Percentage of Deviation of Parameters From The Value of 1 in Each Study.

	*c*>1 (%)	Rachlin-*s* <1 (%)	Green-*s* <1 (%)
Bickel A^SA^	85^***^	93^***^	85^**^
Bickel B^SA^	85^***^	89^***^	81^**^
Bickel C^SA^	81^***^	93^***^	89^**^
Odum^SA^	65	53	65
Cheng Heroin small^SA^	96^***^	98^**^	100^***^
Cheng Heroin large^SA^	80^*^	75^*^	75^*^
Estle 1 small^NC^	70	60	70
Estle 1 large^NC^	80^*^	75^*^	75^*^
Estle 3 small^NC^	81^**^	85^**^	85^**^
Estle 3 medium^NC^	78^**^	74^*^	67
Estle 3 large^NC^	63	59	56
Cheng Control small^NC^	88^***^	91^***^	100^***^
Cheng Control large^NC^	84^***^	89^***^	93^***^
OU^NC^	82^**^	71^*^	71^*^
SA	89^***^	92^***^	90^***^
NC	80^***^	82^***^	79^***^
Total	84^***^	84^***^	86^***^

Note: *p<.05; **p<.01; ***p<.001. SA: percentage computed across all cases in studies with substance abuse individuals; NC: percentage computed across all cases in studies with non-clinical individuals. Total: percentage computed across all cases in all studies. When SA and NC are superscripts, they indicate whether the study contains substance abusers or non-clinical people.

As shown in Table 7, for *c-* and Rachlin-models, 11 out of 14 (78.6%) studies showed that the new parameters were different from 1. For Green-model, 10 out of 14 (71.4%) displayed such a pattern. Binomial test showed that a significant proportion of the values were above or below the target value of 1.

### Relationship Between Estimated Parameters and Discount Tendency

Previous studies using the Rachlin-model and/or Green-model [8], [22]–[24] did not examine a possible association between the parameters, but it may be useful to do so in order to understand their behavior in representing discount tendency. Given the non-normal distributions and the few outliers, and in order to keep the number of data points constant for all models, we adopted Spearman rank correlation for these analyses without deleting observations. Table 8 shows the Spearman rank correlations between the two parameters in each model in each study.

**Table 8:** Spearman Rank Correlations Between the Model Parameters.

	*c*-model (*k,c*)	Rachlin-(*k,s*)	Green-(*k,s*)
Bickel A^SA^	.181	−.239	−.505^**^
Bickel B^SA^	−.161	−.363	−.733^***^
Bickel C^SA^	.204	−.243	−.541^**^
Odum^SA^	−.397	−.162	−.834^***^
Cheng Heroin small^SA^	.266^*^	−.845^***^	−.845^***^
Cheng Heroin large^SA^	−.273^*^	−.977^***^	−.640^***^
Estle 1 small^NC^	−.429	−.442	−.829^***^
Estle 1 large^NC^	−.053	−.439	−.433
Estle 3 small^NC^	−.266	−.447^*^	−.584^**^
Estle 3 medium^NC^	−.041	−.597^**^	−.814^***^
Estle 3 large^NC^	−.410^*^	−.808^***^	−.925^***^
Cheng Control small^NC^	.140	−.903^***^	−.353^**^
Cheng Control large^NC^	.492^***^	−.963^***^	−.374^**^
OU^NC^	0.0	−.822^***^	−.455^*^
SA	.078	−.621^***^	−.494^***^
NC	−.018	−.408^***^	−.583^***^
Total	−.012	−.620^***^	−.555^***^

Note: *p<.05; **p<.01; ***p<.001. SA: correlation computed across cases in studies with substance abusers. NC: correlation computed across all cases in studies with non-clinical individuals. Total: correlation computed across all cases in all studies. When SA and NC are superscripts, they indicate whether the study contains substance abusers or non-clinical people.

As seen in Table 8, there is a general negative relationship between parameters *k* and *s* in Rachlin- and Green-models. A larger *k* in both models led to a greater devaluation whereas a smaller *s* reduced this trend. Theoretical analyses of the function forms (Figure 2) show that *s* regulates the inflection and slope of the curves whereas *k* is more closely related to the general base level of discounting. Thus, the negative correlations between *k* and *s* indicated that these parameters tended to counteract their impact on discounting, allowing the functions to have greater flexibility. In contrast, parameters *c* and *k* in the *c*-model behaved more independently; with *c* determining the base level of discounting and *k* further adjusting the curvature of the subjective value function over time as shown in Figure 1.

In order to further explore the relationship of the observed discounting and the estimated parameters, we used the area under the curve (AUC) as a parameter-free measure of the discounting tendency. AUC is a non-parametric measure adopted by [33], which is based on adding the segments enclosed by the empirically obtained indifference curve. AUC is standardized and thus ranges from 0 to 1. A larger AUC indicates that the participants discount future outcome to a lesser extent, whereas a smaller AUC represents higher discounting. We note that theoretically, the area under a curve is the integral of the function used to represent discounting. This integral depends on the model parameters and in some cases, for Rachlin-model for example, the close form is quite complex. It is beyond of the scope of this paper to present a detailed analysis of such definite integrals, but suffice it to say that in general for these models the integral is inversely proportional to *k*. Thus, to the extent that the models are close approximations to the data, we expect that the relationship between *k* and AUC to be negative.

Table 9 shows Spearman rank correlations between AUC and parameters in each study. Table 9 shows that the *k* parameters in base-model and *c*-model were highly negatively related with AUC. The pattern was more varied for Rachlin- and Green-models. The *k* in Rachlin-model was significantly and negatively related with AUC in 9 out of 14 studies (64.3%). In contrast, *k* was related with AUC in only three studies in the Green-model (21.0%). The parameter *s* was significantly correlated with AUC in 9 studies (64.3%) in each of Rachlin and Green-models. In all, but the heroin and control participants in [30], the significant relationships between *s* and AUC were also negative.

**Table 9:** Spearman Correlations Between AUC and Estimated Parameters.

	Base *k*	c model *k*	*c*	Rachlin-*k*	Rachlin-*s*	Green-*k*	Green-*s*
Bickel A^SA^	−.971^***^	−.718^***^	−.063	−.692^**^	−.410^*^	−.199	−.479^*^
Bickel B^SA^	−.955^***^	−.903^***^	−.075	−.269	−.676^***^	.162	−.662^***^
Bickel C^SA^	−.957^***^	−.958^***^	−.284	−.467^*^	−.665^***^	.087	−.731^***^
Odum^SA^	−.912^***^	−.765^***^	−.044	−.873^***^	−.044	−.345	−.010
Cheng Heroin small^SA^	−.793^***^	−.788^***^	−.485^***^	−.608^***^	.305^***^	−.615^***^	.274^*^
Cheng Heroin large^SA^	−.687^***^	−.736^***^	−.116	−.224	.068	−.209	−.182
Estle 1 small^NC^	−.937^***^	−.973^***^	.355	−.532^*^	−.380	−.098	−.329
Estle 1 large^NC^	−.985^***^	−.983^***^	.015	−.580^**^	−.347	−.302	−.671^**^
Estle 3 small^NC^	−.990^***^	−.940^***^	.081	−.501^**^	−.405^*^	−.194	−.548^**^
Estle 3 medium^NC^	−.992^***^	−.993^***^	−.015	−.299	−.531^**^	.161	−.634^***^
Estle 3 large^NC^	−.979^***^	−.968^***^	.263	−.064	−.466^*^	.244	−.526^**^
Cheng Control small^NC^	−.510^***^	−.807^***^	−.179	−.524^***^	.300^*^	−.563^***^	−.106
Cheng Control large^NC^	−.968^***^	−.789^***^	−.500^***^	−.586^***^	.412^**^	−.600^***^	−.196
OU^NC^	−.993^***^	−.980^***^	−.071	−.207	−.310	.137	−.414^*^
SA	−.779^***^	−.792^***^	−.243^***^	−.405^***^	−.363^***^	−.075	−.462^***^
NC	−.131^*^	−.241^***^	−.121	−.190^**^	−.069	−.047	−.279^***^
Total	−.471^***^	−.442^***^	−.274^***^	−.371^***^	−.059	−.185^***^	−.226^***^

Note: *p<.05; **p<.01; ***p<.001. SA: correlation computed across cases in studies with substance abusers. NC: correlation computed across all cases in studies with non-clinical individuals. Total: correlation computed across all cases in all studies. When SA and NC are superscripts, they indicate whether the study contains substance abusers or non-clinical people.

In order to better understand the independent contribution of the parameters in predicting the discounting behavior as measured by AUC, partial Spearman rank correlations between AUC and a single parameter when controlling for the other were obtained. Table 10 displays these partial correlations.

**Table 10:** Partial Spearman Rank Correlations Between Model Parameters and AUC.

	*c-*model *k*	*c*	Rachlin-*k*	Rachlin-*s*	Green-*k*	Green-*s*
Bickel A^SA^	−.776^***^	−.690^***^	−.892^***^	−.820^***^	−.477^*^	−.630^**^
Bickel B^SA^	−.930^***^	−.520^**^	−.750^***^	−.862^***^	−.635^***^	−.810^***^
Bickel C^SA^	−.959^***^	−.317	−.868^***^	−.908^***^	−.537^**^	−.816^***^
Odum^SA^	−.853^***^	−.588^*^	−.892^***^	−.384^*^	−.639^**^	−.573^*^
Cheng Heroin small^SA^	−.770^***^	−.460^***^	−.688^***^	−.492^***^	−.744^***^	−.580^***^
Cheng Heroin large^SA^	−.804^***^	−.486^***^	−.749^***^	−.735^***^	−.430^**^	−.420^**^
Estle 1 small^NC^	−.972^***^	−.297	−.845^***^	−.811^***^	−.701^**^	−.736^**^
Estle 1 large^NC^	−.984^***^	−.205	−.870^***^	−.823^***^	−.887^***^	−.933^***^
Estle 3 small^NC^	−.955^***^	−.512^**^	−.834^***^	−.812^***^	−.758^***^	−.831^***^
Estle 3 medium^NC^	−.994^***^	−.455^*^	−.906^***^	−.926^***^	−.794^***^	−.879^***^
Estle 3 large^NC^	−.978^***^	−.586^**^	−.847^***^	−.882^***^	−.749^***^	−.814^***^
Cheng Control small^NC^	−.802^***^	−.112	−.618^***^	−.474^***^	−.645^***^	−.393^**^
Cheng Control large^NC^	−.721^***^	−.209	−.772^***^	−.700^***^	−.567^***^	−.740^***^
OU^NC^	−.983^***^	−.362	−.853^***^	−.862^***^	−.063	−.398^*^
SA	−.799^***^	−.298^***^	−.722^***^	−.708^***^	−.521^***^	−.651^***^
NC	−.245^***^	−.129^*^	−.285^***^	−.227^***^	−.183^**^	−.327^***^
Total	−.463^***^	−.311^***^	−.520^***^	−.396^***^	−.383^***^	−.402^***^

Note: *p<.05; **p<.01; ***p<.001. SA: correlation computed across cases in studies with substance abusers. NC: correlation computed across all cases in studies with non-clinical individuals. Total: correlation computed across all cases in all studies. When SA and NC are superscripts, they indicate whether the study contains substance abusers or non-clinical people. When SA and NC are superscripts, they indicate whether the study contains substance abusers or non-clinical people.

Results in Table 10 show that in the *c*-model, the parameter *k* was negatively associated with AUC after controlling for *c*. In contrast, *c* did not reveal a consistent relationship with AUC in the presence of *k*. This is consistent with model analyses (Figures 1) as *c* sets the base level of subjective value independently of delay, whereas *k* affects subjective value via the weighting of delay. In Rachlin- and Green-models *k* and *s* were significantly negatively related with AUC after controlling for the other parameter, which further demonstrated their more complex relationship in determining subjective value as exhibited in Figures 1 and 2. In addition, the net relationship between AUC and *k,* or *s* was also negative in [30]. Hence, in the *c*-model, *k* uniquely described the AUC. Whereas in Rachlin- and Green-models, both *k* and *s* related to AUC with a greater *k* (or *s*) being associated with a smaller AUC (i.e., greater discounting).

### Analysis of the Magnitude Effect

The magnitude effect refers to the phenomenon that greater discounting is observed for smaller than for larger gains [22]. This effect is due to changes in the conditions (payoff level) and provides additional tests of models' parameters. In particular, the parameter *s* has been described as a person measure [24] and hence would not be expected to change with conditions. The *k* parameter of Green- and the base-model has often been used to describe individual differences as earlier described, but it has also been used to describe changes due to situations (e.g., to account for sign and magnitude effects [25], [30]); hence, the meaning of *k* may be broader to account for both person and situation based variability. The *c* parameter is directly indexing a valuation process and hence it is expected to vary with payoff level. We examine these possibilities in tests of the magnitude effect.

We tested the effect in studies that contained a within-subjects manipulation of payoff size: Bickel B (A = 100) vs. Bickel C (A = 1000); Cheng heroin small (A = 200) vs. Cheng heroin large (A = 50000); Cheng control small (A = 200) vs. Cheng control large (A = 50000); Estle 1 small (200) vs. Estle 1 large (A = 40000); Estle 3 small (A = 100) vs. Estle 3 medium (A = 20000); Estle 3 small (A = 100) vs. Estle 3 large (A = 60000); and Estle 3 medium (A = 20000) vs. Estle 3 large (A = 60000). In [28], the magnitude effect was not reported and we found that there was no difference in AUC between condition B (mean AUC = .431) and condition C (mean AUC = .398), paired *t*(26) = .91, *p* = .37. In [30], the authors reported that there was a magnitude effect in both heroin participants and controls. In [22], it was found that the magnitude effect existed in all comparisons listed above except when comparing medium and large magnitudes in Experiment 3.

For studies in which the magnitude effect was not revealed by mean AUC changes, no changes of parameters were observed in any of the tests conducted. For the studies with magnitude effects, Wilcoxon Signed Ranked on parameter values within each study showed that *k* from all models was able to reflect this effect with smaller values in the large than in the small magnitude conditions, all *p*-values ≤.05. In terms of parameter *c*, it reflected the magnitude effect in all studies (*p*-values ≤.01), but in Cheng control participants and Estle 1. Similar to *k*, *c* was smaller in the large than in the small magnitude conditions. Note that *c* can be expressed in terms of *a* in the *a*-model and thus it is of particular interest to analyze whether the *a* parameter is constant, or whether it changes with magnitude depicting intrinsic changes in subjective valuation due to payoff size. We proceeded to analyze the magnitude effect in terms of the parameter *a* by first estimating it at each curve level and confirming its relationship with *c*. Wilcoxon Signed Ranked test on parameter *a* for each study showed that *a* indexed the magnitude effect in all studies with a smaller *a* in the small magnitude conditions, all *p*-values <.05. Parametric tests (paired samples *t*-test) on mean parameter values replicated these results with the following exceptions: *k* parameter of Green-model did not show an effect in [30]; *a* parameter did not show an effect in the Estle 1 study.

In terms of *s*, using Rachlin-model the changes were sometimes in the direction of the magnitude effect (i.e., smaller *s* in the large than the small payoff condition) but other times the change was in the opposite direction. In Estle 3 (small vs. large; small vs. medium), the parameter *s* showed an opposite magnitude effect: *s* was greater in the large than in the small magnitude conditions, *p*-values <.05. There was no change of *s* in Estle 1 between the two magnitude conditions, *p-*value>.1. In Cheng control and heroin groups the direction of the magnitude effect was not consistently shown by parameter *s*, and the parameter values did not change significantly.

Parameter *s* in Green-model showed that it changed significantly with magnitude in both Cheng heroin participants and control participants in the direction of the effect, *p*-values <.01. The change was in the opposite direction in Estle 3 when comparing the small and large magnitude conditions, *p*<.05, but no effect when comparing the small and medium conditions. Similarly, there was no effect of *s* in Estle 1 when comparing small and large conditions.

Taken together, *k*, *c* and *a*, varied consistently in the direction of the magnitude effect with smaller values in conditions with larger A. In contrast, *s* did not show a consistent pattern and this may be in agreement with the expectation that *s* must remain constant because it was to reflect an individual level tendency [24]. In order to further clarify the extent to which each parameter moved in the predicted direction, we computed the effect size (Cohen's *d*) in each study. Following the methods provided by [34], weighted average *d* across the studies were obtained along with its 95% confidence interval. The results appear in Table 11.

**Table 11:** Meta-Analysis of Effect Size (Cohen's d) When Testing Magnitude Effect.

Parameters	Avg(*d*)	SD(*δ* ^a^)	95% CI
*c*-model *k*	0.851	0.472	[.415–1.29]
* c*	0.312	0.141	[.132–.492]
*a*-model *k*	0.861	0.53	[.377–1.35]
* a*	0.616	0.273	[.343–.899]
Rachlin -*k*	0.39	0.0	[.283–.497]
Rachlin-*s*	−0.087	0.529	[−.569–.395]
Green-*k*	0.317	0.035	[.184–.450]
Green-*s*	−0.008	0.674	[−.613–.597]

Note. a: According to Hunter & Schmidt (2004), the population variance of effect size (*δ^2^*) is obtained by subtracting variance due to sampling error from observed variance adjusted by sample size.

Table 11 shows that the 95% CIs include the value of zero only for the effect of the *s* parameter in both Rachlin- and Green-models. Although the sample size made the conclusions from this meta-analysis tentative (we have five studies demonstrating the magnitude effect), it was safe to say that there was no evidence of a consistent magnitude effect with parameter *s*. This lends support to the assumption that *s* should remain constant with changes in contexts, but further studies are needed to clarify its role in describing context effects.

### Parameter Comparison between SA and NC in an Experimental-Control Design

The groups of SA and NC participants come from different studies and conditions; therefore direct comparisons of parameter values is limited. Among these studies, however, [30] has both heroin-dependent patients and controls, and the groups were globally matched on age, gender, education, and income (for more details refer to [30]). Focusing on participants in this study, we compared the parameter values keeping study conditions separate (i.e., small and large magnitude conditions). The mean and median parameter values are found in Table 6, but to facilitate reading of the results, we present them in Table 12.

**Table 12:** Median and Mean Values of the Parameters of Heroin and Control Participants in [30].

		*c*-model		Rachlin-model		Green-model	
		*k*	*c*	*k*	*s*	*k*	*s*
Heroin^S^	Median	.008 (.007)	1.276 (.35)	.117 (.24)	.512 (.27)	.122 (.72)	.333 (.17)
	Mean	.010 (.01)	1.527 (.91)	.270 (.43)	.556 (.19)	1.965 (5.25)	.382 (.20)
Heroin^L^	Median	.002 (.002)	1.151 (.23)	.051 (.11)	.520 (.23)	.047 (.23)	.286 (.13)
	Mean	.002 (.001)	1.292 (.39)	.153 (2.7)	.539 (.32)	.754 (3.25)	.260 (.12)
Control^S^	Median	.004 (.002)	1.040 (.07)	.012 (.02)	.808 (.20)	.009 (.01)	.653 (.22)
	Mean	.005 (.004)	1.088 (.16)	.043 (.01)	.789 (.17)	.187 (.95)	.586 (.23)
Control^L^	Median	.001 (.0004)	1.061 (.06)	.008 (.01)	.663 (.20)	.005 (.005)	.381 (.19)
	Mean	.0009(.0005)	1.067 (.06)	.014 (.02)	.753 (.27)	.008 (.06)	.535 (1.2)

Note: Superscripts ^S^ and ^L^ index small and large payoffs. Numbers in parentheses are IQR for median, and standard deviation for means; means computed without outliers.

In terms of median *c* and *k*, they were larger in SA than in NC in both small and large magnitude conditions, both Mann-Whitney test *p*-values <.01. In Rachlin- and Green-models *k* was larger in SA than in NC, and the reverse was true for *s* in all conditions, all Mann-Whitney test *p*-values <.01. Therefore, the parameter *k* in all models indexed the difference between SA and NC in both magnitude conditions; so did the *c* parameter and both parameters moved in the expected direction, i.e., they were smaller in NC participants. Parameter *a* from *a*-model performed similar to parameter *c*: *a* was significantly smaller in SA than in NC. The *s* parameter, on the other hand, moved in the opposite direction with smaller values for the SA individuals. As seen in Figure 2, greater *s* implies greater discounting which would be expected for the SA participants instead.

There are limitations to using non-parametric tests when making comparisons because the magnitude of the quantities being compared is lost. Hence we performed tests of mean differences between the SA and NC groups. In order to conduct these tests, we identified the outliers by the procedure earlier described. We also assessed departures from symmetry of the distributions and transformed them using the natural log function when needed.

Independent *t*-tests were performed for each parameter between heroin-dependent and the non-clinical groups. For *c*-model, parameter *k* was larger in SA than in NC in the small magnitude condition, *t*(110) = 5.24, *p*<.001, and in the large magnitude condition, *t*(109) = 5.17, *p*<.001. Parameter *c* was also larger in SA in both small and large magnitude conditions, *t*(110) = 5.11, *p*<.001 and *t*(109) = 5.0, *p*<.001, respectively.

For Rachlin-model, parameter *k* demonstrated the group difference in both magnitude conditions, *t*(110) = 7.20, *p*<.001, and *t*(104) = 7.38, *p*<.001, respectively. Parameter *s*, however, was smaller in SA than in NC in small and large magnitude conditions, with *t*(110) = −7.20, *p*<.001 and *t*(110) = −5.28, *p*<.001, respectively.

Similar to the other models, in Green-model, SA displayed a larger *k* when compared to NC, *t*(107) = 3.42, *p* = .001 in the small magnitude condition; *t*(105) = 2.38, *p*<.05 in the large magnitude condition. Parameter *s* moved in the opposite direction of parameter *k*. In each case, *s* was smaller in SA: *t*(104) = −6.024, *p*<.001 in the small condition; *t*(110) = −4.013, *p*<.001 in the large condition. Thus, in agreement with the non-parametric tests the parameters *k* and *c* consistently represented the group differences whereas the parameter *s* did not.

## Discussion

The present study aimed to better understand the basis of the observed discounting behavior of both substance abusers and non-clinical individuals. Traditionally the single parameter model of Equation 1 has been used to describe group differences with parameter *k* being on average greater for the substance abusers than for the non-clinical individuals. Our results replicate these findings. However, the *k* parameter is too general and differences in discounting tendency may be due to valuation process differences, or to time perception distortions that in turn affects the subjective value of a future amount. The present study sought to clarify the psychological basis for discounting in intertemporal choice and the observed group differences using a modeling approach. The models tested represent valuation, time perception, and general time/amount scaling functions: *c*-, Rachlin-, and Green-models, respectively. Rachlin- and Green-models have been widely used with substance abusers and hence served as benchmarks for investigating individual differences. The *c*-model, as it derives from the *a*-model, has been theoretically considered but not tested. This study advances our understanding of the manner in which models of discounting behavior represent different psychological process for different individual and conditions.

The analyses first elucidated the similarities and differences of the function forms and how model parameters produce varying discounting tendencies for different A and D levels. Through such simulations, it was shown that Rachlin- and Green-models predicted very similar discounting for a wide array of delays. In contrast, the base-model, and *c*-model with greater *c*>1, can produce deeper discounting patterns than the Rachlin- and Green-models at a fixed *k*. Furthermore, the analyses demonstrated the different roles of *k* in the different models. In *c*-model, *k* had a greater influence in regulating the shape of the discounting curve, whereas *c* set the overall base-level of discounting. In contrast, in Rachlin- and Green-models, parameter *s* appeared to regulate the changes of subjective value over time with *k* serving to set the base level of discounting. However, the relationship between *k* and *s* was very interactive; a large *s* could counteract a small *k* and vice versa. Results of the simulation also demonstrated that A affected the rate of discounting (change in subjective value per unit of time).

Moreover, discounting rate is not constant throughout the delay periods and this implies that individuals are more sensitive to delay differences closer to the present moment than later. Tests of such implications are needed to further study the models' validity. For example, assume a participant has estimated *k* = 0.08 and *s* = 0.5 and let us use the Rachlin-model without loss of generality. The model predicts a subjective value of 798.09 for the option (A = 1000, D = 10) and a value of 736.5 for (A = 1000, D = 20); this is a 61.59 drop in subjective value of A in a period difference of 10 D units. In later periods (between D = 20 and D = 30) the drop in subjective value of A is of 41.17. This implies that the preference for the sooner option would be stronger in the first than in the second comparison. However, it is likely that by dominance a decision maker confronted with such pairs of options would select the sooner options with 100 percent certainty in both situations; thus show no reduced time sensitivity in choice. This means that the models' predicted preferences may not agree with participants' behavior once the models are tested with another method. More generally, cross-validation studies are much needed, and by this we mean that a function derived via the indifference point methodology is then used to predict behavior outside of the data employed in model fitting.

In terms of model testing, the analysis inspected not only model fit and the corresponding proportion of variance accounted for, but also examined the meaning of the estimated parameters in different groups and in different magnitude conditions. Ultimately, it is the meaning of such parameters that can provide psychological understanding. First, compared to the base-model, all the two-parameter models improved fit non-trivially as demonstrated with the SBC measure and with the tests of mean and median parameter values against the default value of 1. Among the two-parameter models, Rachlin-model displayed the best fit at the individual level in both SA and NC. This result contrasts with that of [8], [23] finding that no difference was found between Rachlin- and Green-models. A possible reason for this difference was that a greater number of studies were included, with a wider range of D. We note, however, that all of the models performed extremely well when considering *R^2^*, and that the function forms tend to overlap in many situations as shown in the function form analysis. Thus, not finding differences may be more the rule than the exception.

In terms of estimated parameters, previous research found that the parameter *s* in Green- and Rachlin-models was smaller than 1 [23]. We replicated this finding in fourteen studies. Additionally, we found that a significant proportion of cases had a *c* larger than 1 in *c*-model. Therefore, from the perspective of whether the two-parameter models may be easily reduced to the one-parameter base model, our results indicated that this was not the case. Psychologically, in terms of the valuation process, *c* being larger than 1 implies a power function for subjective value that is concave (i.e., *a*<1 in *a*-model). This is consistent with the general value function of prospect theory and other utility functions that assume diminishing returns [15]. That the parameter *s* was also not equal to 1 echoed the notion that subjective time was different from objective time [19]–[21]. However, we note that having *s*<1 meant that time was not enhanced, but rather shrunk, and the fact that *s* was smaller in SA than in NC implied that the subjective delays were smaller for SA than NC. This contradicts the expectation that SA's would overestimate the duration of time as reported in [20]. Indeed, more generally *s*<1 reduces discounting as observed in Figures 1 and 2, everything else constant. This means that the greater discounting observed in SA cannot be attributed to time perception processes in the manner represented by *s* in Rachlin- and Green-models. It is the compensation of a smaller *s* by a greater *k* that accounts for greater discounting in the SA individuals.

Another finding was that Rachlin- and Green-models produced estimated parameters that were statistically and negatively related to each other. This suggests that the models improved fitting in part by having parameters compensated for each other. As the function form analyses showed, the parameter *s* could counteract the effects of *k* and vice versa. This was not so for the parameter *c* in the *c*-model. Furthermore, the models' parameters showed that the parameters *k* and *s* were negatively related to AUC when controlling for the other, whereas this was not the case for *c*. In combination, this shows that *c* is a less redundant parameter.

Additional analyses on contextual differences showed that the parameter *k* in all models was consistently greater in the small versus the large magnitude conditions, thus being able to describe the *magnitude effect*. The same was true in almost all cases for the *c* parameter and in all cases for the corresponding *a* parameter. In contrast, parameter *s* in either the Rachlin- or the Green-model did not show a consistent pattern in relationship to the magnitude effect. These results reestablish that the *k* parameter may be conceived as a situation specific, or context effect index, but not *s*.

In a similar vein, when testing models with data from heroin patients and control participants within a single study, *k* was larger in the heroin group than in controls. This was also true for the *c* parameter. In contrast *s* was smaller for the substance abuse individuals than the non-clinical group. Hence, parameters *k* and *c* systematically measured the average individual tendencies, whereas *s* allowed for better model fit but its values did not advance additional meaning that could describe, or explain these person level effects.

Overall, we found that Rachlin-model provided the best description of the data across studies from the perspective of variance accounted for, followed by Green-model. This indicated that a time perception function (Rachlin-model), or a general amount-to-time scaling function (Green-model) was beneficial to the base model in explaining additional variance. However, both models have challenges with regards to the interpretation of their parameters. In particular, the finding that the SA individuals had *s* values that would result in less rather than greater discounting is problematic; model fitting was significantly improved with *s*, but psychological understanding was not. Furthermore the *s* parameter negatively correlated with *k* which resulted in small changes in *s* that counteracted the movements of *k* across the many studies reviewed. Thus, *s* adds flexibility to the models, but it does not add explanatory power to them. Further studies are needed to establish *s* as a person level index. In terms of *k*, it described both context and person level effects well, but this success is also its weakness as it does not uniquely represent either factor.

In terms of *c*-model, it also performed better than the base-model. Its parameter *c* varied with magnitude and revealed group differences in the expected direction (i.e., greater *c* for the SA than the NC groups), and it tended to do so independently of *k* as correlation analysis showed. Hence, *c*-model may be a viable descriptive and explanatory model of the general discounting tendencies observed between SA and NC. Nonetheless, this model is not free of issues altogether. *c*>1 implied that individuals had a marginally decreasing utility function, and this was confirmed upon estimation of the parameter *a*. Analyses of the magnitude effect revealed that *a* varied as a function of magnitude, which positioned *a* as a weight on outcome. It is not entirely clear why such weighting would change with levels of payoffs; one possibility may be that individuals pay greater attention to larger quantities. If so, *a* is not an index of a person level trait.

An alternative interpretation of *c*>1 is that the delay D is enlarged by a subjective additional delay as shown in the equivalent form, *c* = 1+*kd*, with *d* representing additional time units added to the denominator of the base-model, denominator  = 1+*k*(*d*+D). In this re-expression, the greater discounting by substance abusers can be attributed to an increased subjective delay that stems from money not changing fast enough to their most preferred reinforcer-drugs. The secondary nature of money as a reinforcer may not always depend on internal individual proclivities. For example, people suffering from hyperinflation in Zimbabwe were shown to purchase things as soon as they received their salary [35]. In late 1980s in China, inflation was extremely high due to the economic structural reform (21.3 percent in 1988 and 16.0 percent in 1989) and a survey conducted on 150,000 urban households during that period reported that consumers had strong motives to transfer savings to products [36]. Irrespective of whether the additional discounting is internally or externally determined, the *c*-model allows for a positive shift in delay that reduces subjective value. This conclusion demands further empirical studies and highlights the inability of the current data to provide a clear meaning of the parameter *c* (or *a*). As is true for *k*, the *c* parameter did not reveal itself as a pure person or task level measure.

In sum, the present study replicated the finding that known models (base-, Rachlin-, and Green-models) could describe indifference curves derived from standard intertemporal choice staircase or titration procedures extremely well. In particular, Rachlin-model appeared as the most successful one in terms of model fitting. The *k* parameter across all models was able to consistently describe both situation and person dependent variability in discounting. In addition, we showed that a value based model, *c*-model (or *a*-model), also provided excellent fit and its parameters also tracked group and task differences. But the descriptive ability of a model does not guarantee its explanatory power. Because the *k* parameter was so essential to representing both group and context differences across the models, it remains unclear whether value or time perception processes lead to greater discounting. In addition, the ambiguity of *k* as both a person and situation based index demands further research to unpack its meaning. The same is true for the *c*-model which advances interpretations of *c* as a weight on the nominal amount A, via the *a*-model, or as an additional subjective delay that reflects the secondary reinforcing nature of money. These interpretations are quite different, thus necessitating further research. Additionally, the results showed that the *s* parameter was perhaps too volatile to render a clear meaning. It is also not known if *s* is time-unit dependent. For example, many studies present delays in one unit to the participants (e.g., months) but then transform them to another unit for hyperbolic model fitting (e.g., days). It is unclear whether such transformations improve model fitting but at the expense of understanding time perception via the power function with *s*.

Additional considerations pertain to the methodology used to derive hyperbolic models. The indifference point methodology typically presents participants with an immediate smaller option versus a delayed larger one to derive the function. However, participants are typically not asked to compare binary options defined by payoffs and delays at other combinations of delays to further assess the predictive validity of the derived function. A vast literature in preferential choice has demonstrated that individuals vary their preferences among options depending on the method and conditions that are used to assess them [37]–[39]. Thus, it stands to test whether the implied changes in subjective value per unit of time derived from the indifference point methodology can predict choices that require the decision maker to directly compare binary choice options with varying monetary and delay differences. Indeed, using a general choice paradigm researchers [40]–[42] have begun to show limitations of the base- hyperbolic model. In addition, we point that the hyperbolic functions tested are silent to the units of delay making the models incomplete from the perspective of being full psychological accounts of how individuals contrast monetary versus delay differences. As earlier stated, for any given model, the prediction would be the same if D were days or D were years, but clearly these units would have great effect on individuals' discounting.
